# Dietary Tryptophan Requirement of Juvenile Hybrid Grouper (*Epinephelus fuscoguttatus*♀ *× E. lanceolatus*♂)

**DOI:** 10.3390/ani15010104

**Published:** 2025-01-05

**Authors:** Jiaxian Chen, Xiaohui Dong, Qihui Yang, Shuyan Chi, Shuang Zhang, Beiping Tan, Junming Deng

**Affiliations:** College of Fisheries, Guangdong Ocean University, Zhanjiang 524088, China; jiaxianchen11@163.com (J.C.); dongxiaohui2003@163.com (X.D.); qihuiyang03@163.com (Q.Y.); chishuyan77@163.com (S.C.); zhangshuang198610@126.com (S.Z.)

**Keywords:** hybrid grouper, tryptophan, growth, protein metabolism

## Abstract

A 10-week feeding study was conducted to examine the effects of dietary tryptophan (Trp) levels on the growth performance and protein metabolism of hybrid grouper (*Epinephelus fuscoguttatus*♀ × *E. lanceolatus*♂) with the aim of determining the optimal Trp requirement. Six isonitrogenous and isolipidic experimental diets were formulated, containing varying levels of Trp (0.26%, 0.32%, 0.42%, 0.46%, 0.58%, and 0.62% of diet). Each diet was administered to 90 hybrid groupers with an initial average weight of 10.52 ± 0.02 g. Dietary levels of 0.32–0.62% Trp generally promoted growth performance and feed utilization in hybrid grouper, with the 0.46% Trp level demonstrating the highest weight gain and protein efficiency ratio. Furthermore, fish fed with 0.46% Trp exhibited higher activities of intestinal lipase and amylase compared to other groups. Furthermore, the activities of Trp metabolism-related enzymes in the liver were markedly elevated by this Trp level, including indoleamine 2,3-dioxygenase, 5-hydroxytryptophan decarboxylase, and tryptophan-2,3-dioxygenase. Additionally, a dietary level of 0.46% Trp resulted in a substantial increase in the activities of protein metabolism-related enzymes such as adenosine monophosphate deaminase and glutamate dehydrogenase in the liver. Dietary levels of 0.32–0.46% Trp activated the hepatic mammalian target of rapamycin pathway, while a dietary 0.46% Trp level inhibited the hepatic amino acid response signaling pathway. In summary, suitable dietary Trp levels enhanced intestinal digestive enzyme activities, facilitated both protein synthesis and catabolism, and ultimately improved growth performance in hybrid grouper. Analysis using a second-order regression curve of weight gain and protein efficiency ratio indicated that the optimal dietary Trp requirement for hybrid grouper ranged from 0.41–0.46% of diet (0.82–0.92% of dietary protein).

## Simple Summary

The present study investigated the impact of various levels of tryptophan on the growth performance and protein metabolism of hybrid grouper (*Epinephelus fuscoguttatus*♀ × *E. lanceolatus*♂). Six experimental diets were formulated to be isonitrogenous (50% of dry diet weight) and isolipidic (12% of dry diet weight), containing varying concentrations of tryptophan (0.26%, 0.32%, 0.42%, 0.46%, 0.58%, and 0.62% of diet). Utilizing a second-order regression curve analysis of weight gain and protein efficiency ratio, the optimal dietary tryptophan requirement for hybrid grouper ranged from 0.41% to 0.46% of diet (0.82–0.92% of dietary protein). The suitable level of tryptophan in the diet was observed to enhance intestinal digestive enzyme activity, stimulate protein synthesis and catabolism, consequently enhancing the growth performance of hybrid grouper.

## 1. Introduction

Tryptophan (Trp) is recognized as an essential amino acid that is crucial for normal growth and maintaining homeostasis in fish. Recent research has highlighted the multifaceted role of Trp, not only in protein synthesis but also as a metabolically active amino acid. Trp can undergo hydroxylation and decarboxylation processes, resulting in the production of serotonin, N-acetylserotonin, melatonin, anthranilic acid, and ammonia. Studies have elucidated the significance of Trp, particularly as a precursor to serotonin, in the physiological processes of fish [1]. Serotonin is subsequently converted into melatonin through the enzymatic actions of 5-hydroxytryptamine-N-acetyltransferase and hydroxyindole-O-methyltransferase. In teleost fish, melatonin plays a role in various physiological processes, including immunity, stress response, gut motility, and feed intake [2]. Furthermore, an insufficiency of Trp can lead to growth retardation and various conditions such as scoliosis or lordosis in certain salmon species [3,4].

As one of the ten essential amino acids for fish, Trp must be sourced externally to meet the nutritional requirements of teleost fish. The Trp content in feed ingredients exhibits considerable variability. Notably, Trp level is relatively high in wheat flour, barley flour, wheat gluten, cottonseed meal, blood meal, and fish meal, while being comparatively lower in corn, hydrolyzed feather meal, meat and bone meal, and corn gluten [5]. The specific Trp requirement differs among various aquaculture species. For example, Indian catfish (*Heteropneustes fossilis*) require 0.24–0.27% of diet (0.63–0.71% of dietary protein) [6], while Japanese flounder (*Paralichthys olivaceus*) necessitate 0.50% of diet (1.0% of dietary protein) [7]. Additionally, channel catfish (*Ictalurus punctatus*) require 0.12% of diet (0.50% of dietary protein) [8], rainbow trout (*Oncorhynchus mykiss*) need 0.25% of diet (0.40% of dietary protein) [9], and rohu (*Labeo rohita*) require 0.37% of diet (0.90% of dietary protein) [10]. Research indicated that the optimal inclusion level of Trp in fish feed ranges from 0.12–0.50%.

Hybrid grouper (*Epinephelus fuscoguttatus*♀ × *E. lanceolatus*♂) is a hybrid species resulting from the crossbreeding of saddleback grouper (*E. lanceolatus*) and brown-marbled grouper (*E. fuscoguttatus*). This species is a prominent marine aquaculture candidate in China, distinguished by its fast growth rate, strong resistance to diseases, and notable economic importance. Previous studies have reported optimal dietary requirements for several essential amino acids in hybrid grouper, including lysine [11], methionine [12], arginine [13], threonine [14], leucine [15], and isoleucine [16]. To date, no research has been conducted on the Trp requirements of hybrid grouper. Consequently, this study aimed to evaluate the effects of various dietary levels of Trp on the growth performance and protein metabolism of hybrid grouper, with the objective of determining the optimal dietary Trp requirement.

## 2. Materials and Methods

### 2.1. Experimental Diets

Six isonitrogenous (50% of dry diet) and isocaloric (12% of dry diet) experimental diets were formulated with the addition of coated Trp to achieve specific Trp levels, along with coated alanine to maintain nitrogen balance. The dietary Trp content was set at 0.26%, 0.32%, 0.42%, 0.46%, 0.58%, and 0.62%. The other amino acid composition of diets was adjusted to align with the muscle amino acid profile of hybrid grouper [17]. The formulation and nutritional levels of the experimental diets were presented in Table 1, while the amino acid composition was detailed in Table 2. The amino acid profiles were measured using an automatic amino acid analyzer (Hitachi L8900; Hitachi, Tokyo, Japan) after acid hydrolysis using 6 N HCl at 110 °C for 22 h.

The dietary ingredients were ground and sieved through a 60-mesh screen. Then, the ingredients were weighed according to the specified formula and thoroughly mixed. Guar gum and crystalline amino acids were mixed with water at 40 °C to form a mixture, which was then added to the pre-mixed ingredients along with fish oil, soybean oil, and soy lecithin, followed by thorough mixing. The resulting mixture was passed through a 40-mesh sieve to ensure even distribution of the oil and encapsulated crystalline amino acids. An appropriate amount of distilled water (approximately 15–20%) was added to form a dough. After sieving and mixing evenly, the mixture was processed into pellets using a twin-screw extruder (F-26; South China University of Technology, Guangzhou, China). All feed pellets are air-dried under natural circumstances, sealed, and kept at −20 °C [18].

### 2.2. Experimental Fish and Feeding Management

Juvenile hybrid groupers were acquired from the Hengxing 863 breeding base, and the experiment was conducted at the National Project Marine Aquaculture Seed Engineering Southern Base of Zhanjiang Hengxing Southern Ocean Technology Co., Ltd. (Zhanjiang, China). After a two-week acclimation period in a 0.5 m^3^ indoor tank, hybrid grouper juveniles with an initial average weight of 10.52 ± 0.02 g were randomly divided into 18 tanks (0.5 m^3^), with 30 fish per tank and three biological replicates for each group. The fish were fed twice (7:30 and 17:00) daily until they were visibly satiated. Throughout the experiment, water temperature was kept within the range of 27–30 °C, pH level was maintained between 7.7 and 8.0, ammonia nitrogen concentration was kept below 0.2 mg/L, and dissolved oxygen level exceeded 5.0 mg/L. The rearing trial lasted for a duration of 10 weeks.

### 2.3. Sample Collection

At the conclusion of the feeding trial, all fish were fasted for 24 h and anesthetized with MS-222 (Sigma-Aldrich Co., St. Louis, MO, USA; 0.1 g/L). Subsequently, each tank containing fish was counted and weighed. From each tank, three fish were randomly selected for whole-body composition analysis. Additionally, three fish from each tank were collected to measure body length and to weigh the entire fish, viscera, and liver [15]. Blood samples were collected from five fish per bucket using a 1-mL syringe to withdraw blood from the caudal vein. These blood samples were allowed to stand overnight in a refrigerator at 4 °C before being centrifuged at 3000 rpm for 10 min at 4 °C. The supernatant was then collected and kept at −80 °C. Another six fish per tank were dissected on ice, and the liver and foregut were separated, stored in liquid nitrogen, and then stored at −80 °C.

### 2.4. Measurement Methods

#### 2.4.1. Proximate Composition of Feed and Fish

The proximate composition of both feed and fish body was conducted using the AOAC [19] methods. Specifically, the moisture content was ascertained after the samples were dried to an invariant weight in a 105 °C oven. Crude protein was quantified using a Dumas nitrogen analyzer (Primacs SN100; Skalar, Amsterdam, The Netherlands). Crude fat was extracted through Soxhlet extraction using petroleum ether, while crude ash content was determined after incineration at 550 °C for 8 h.

#### 2.4.2. Digestive Enzyme Activity

Crude enzyme extracts from the foregut were prepared using the methods described by Wang et al. [20] and Castillo-Lopez et al. [21]. Briefly, the foregut was accurately weighed, and nine times the volume of physiological saline was added at a weight-to-volume ratio of 1:9 (g: mL). The resulting mixture was homogenized using a high-throughput tissue homogenizer (Scientific-48L; Ningbo Zhejiang Keqiang Biotechnology Co., Ltd., Ningbo, China) at 4 °C. The homogenate was then centrifuged at 4 °C for 10 min at 3000 rpm using a high-speed refrigerated centrifuge (M1324R; Shenzhen Reward Life Technologies Co., Ltd., Shenzhen, China). The supernatant was collected for subsequent analyses. The activity of trypsin (No. ML036384) was assessed by the double-antibody sandwich method using an ELISA kit (Shanghai Enzyme-linked Biotechnology Co., Ltd., Shanghai, China) following the manufacturer’s instructions. Meanwhile, amylase (No. C016-1-2) and lipase (No. A054-2-1) activities were measured by the microplate method using commercial kits (Nanjing Jiancheng Bioengineering Institute, Nanjing, China). Enzyme activity was expressed as units per gram/milligram of protein. The foregut protein content (No. A045-4-2) was determined using the BCA method using a commercial kit (Nanjing Jiancheng Bioengineering Institute, Nanjing, China).

#### 2.4.3. Serum Biochemical Indices

Serum biochemical parameters, including total amino acids (TAA, No. A026-1-1), blood urea nitrogen (BUN, No. C013-2-1), aspartate aminotransferase (AST, No. C010-2-1), and alanine aminotransferase (ALT, No. C009-2-1), were measured by the colorimetric method, the urease method, and the microplate method using commercial kits (Nanjing Jiancheng Bioengineering Institute, Nanjing, China) following the manufacturer’s instructions, respectively.

#### 2.4.4. Tryptophan and Protein Metabolism-Related Parameters

The liver was accurately weighed; the enzyme extraction process is consistent with that of the foregut. The supernatant was collected for subsequent measurements. The activities of glutamate dehydrogenase (GDH, No. ML669716), 5-hydroxytryptophan decarboxylase (5-HTPDC, No. ML941770), tryptophan hydroxylase (TPH, No. ML969964), tryptophan 2,3-dioxygenase (TDO, No. ML663584), adenosine monophosphate deaminase (AMPD1, No. ML965741), and indoleamine 2,3-dioxygenase (IDO, No. 295630) in the liver were determined by the double-antibody sandwich method using ELISA kits (Shanghai Enzyme-linked Biotechnology Co., Ltd., Shanghai, China). Additionally, the activities of AST (No. C010-2-1) and ALT (No. C009-2-1) were assessed by the microplate method using assay kits (Nanjing Jiancheng Bioengineering Institute, Nanjing, China) following the provided instructions. Enzyme activities were given as units per gram/milligram of protein. The liver protein content (No. A045-4-2) was determined using the BCA method using a commercial kit (Nanjing Jiancheng Bioengineering Institute, Nanjing, China).

#### 2.4.5. Total RNA Extraction, Reverse Transcription, and Real-Time Quantitative PCR Analysis

Total RNA was extracted from the liver using the TransZol Up Plus RNA kit (TransGen Biotech Co., Ltd., Beijing, China) following the manufacturer’s instructions. Subsequently, total RNA from each group served as a template for reverse transcription, utilizing the Evo M-MLV RT Kit II (Accurate Biotechnolgy Co., Ltd., Hunan, China). The reaction system (20 μL) comprised 10 μL of genomic DNA removal product, 1 μL of the Evo M-MLV RT enzyme mixture, 1 μL of the RT primer mixture, 4 μL of 5X RT enzyme reaction buffer mix II, and 4 μL of RNase-free dH_2_O. Real-time quantitative PCR was performed on a LightCycler 480 system (Roche Applied Science, Basel, Switzerland), using β-actin as an internal reference and the SYBR^®^ Green Pro Taq HS Premix II qPCR kit (Accurate Biotechnolgy Co., Ltd., Hunan, China). The thermal cycling program included an initial denaturation at 95 °C for 30 s, followed by 40 cycles of denaturation at 95 °C for 5 s and annealing/extension at 60 °C for 30 s. The reaction system (10 μL) consisted of 5 μL of 2X SYBR^®^ Green Pro Taq HS Premix II, 0.5 μL each of forward and reverse primers, 1 μL of cDNA, and 3 μL of RNase-free dH_2_O. The primers for the target genes used in qPCR were detailed in Table 3, with amplicon sizes ranging from 80 to 200 bp. Standard curves were made with five different dilutions (in triplicate) of the cDNA samples, and amplification efficiency was analyzed according to the following equation: E = 10^(−1/slope)^ − 1. The 2^−∆∆Ct^ technique was used to compute the genes’ relative expression levels [22].

### 2.5. Calculation

$$\mathrm{Weight}\;\mathrm{Gain}\;\mathrm{Rate}\;\left(\mathrm{WGR},\;\%\right)=100\times \frac{\mathrm{Wf}-\mathrm{Wi}}{\mathrm{Wi}}$$

$$\mathrm{Specific}\;\mathrm{Growth}\;\mathrm{Rates}\;(\mathrm{SGR},\;\%/\mathrm{day})=100\times \frac{\mathrm{lnWf}-\mathrm{lnWi}}{d}$$

$$\mathrm{Survival}\;\mathrm{Rate}\;\left(\mathrm{SR},\;\%\right)=\frac{\mathrm{final}\;\mathrm{number}}{\mathrm{initial}\;\mathrm{number}}\times 100$$

$$\mathrm{Feed}\;\mathrm{Intake}\;\mathrm{Ratio}\;\left(\mathrm{FIR},\;\%\right)=100\times \frac{\mathrm{feed}\;\mathrm{consumption}}{[\left(\mathrm{Wi}+\mathrm{Wf}\right)/2]\times d}$$

$$\mathrm{Feed}\;\mathrm{Conversion}\;\mathrm{Ratio}\;\left(\mathrm{FCR}\right)=\frac{\mathrm{feed}\;\mathrm{consumption}}{\mathrm{Wf}-\mathrm{Wi}}$$

$$\mathrm{Protein}\;\mathrm{Efficiency}\;\mathrm{Rate}\;(\mathrm{PER})=\frac{\mathrm{Wf}-\mathrm{Wi}}{\mathrm{protein}\;\mathrm{intake}}$$

$$\mathrm{Condition}\;\mathrm{Factor}\;\left(\mathrm{CF},\;g/{\mathrm{cm}}^{3}\right)=100\times \frac{\mathrm{body}\;\mathrm{weight}(g)}{\mathrm{body}\;\mathrm{length}\;({\mathrm{cm})}^{3}}$$

$$\mathrm{Viscerosomatic}\;\mathrm{Index}\;\left(\mathrm{VSI},\;\%\right)=100\times \frac{\mathrm{viscera}\;\mathrm{weight}(g)}{\mathrm{whole}\;\mathrm{fish}\;\mathrm{weight}\;(g)}$$

$$\mathrm{Hepatosomatic}\;\mathrm{Index}\;\left(\mathrm{HSI},\;\%\right)=100\times \frac{\mathrm{hepatic}\;\mathrm{weight}(g)}{\mathrm{whole}\;\mathrm{fish}\;\mathrm{weight}\;(g)}$$

where Wi and Wf represent the initial and final body weight (g), respectively, and d represents the number of experimental days.

### 2.6. Statistical Analysis

The normality test was performed using the Shapiro–Wilk method, and the data satisfied the normal distribution when *p* > 0.05. The homogeneity test was performed using the Levene test, and the data were satisfied when the homogeneity was *p* > 0.05. Data were analyzed using SPSS 25.0 through one-way analysis of variance (ANOVA), followed by Duncan’s test for comparing means among different treatment groups. Furthermore, linear and quadratic orthogonal polynomial contrasts were utilized to assess responses for all dependent variables across varying levels of dietary Trp. The adjusted R^2^ (Adj. R^2^) was calculated as previously described by Kvålseth [23]. Statistical significance was established at *p* < 0.05.

## 3. Results

### 3.1. Growth Performance and Body Index

As the dietary Trp level increased, significant differences (*p* < 0.05) were observed in the FBW, WGR, SGR, PER, and VSI (Table 4). These parameters exhibited a trend of first increasing and then decreasing, with the highest values observed at a dietary Trp level of 0.46%. In contrast, no statistically significant differences were found in the FCR, FIR, CF, and HSI across the dietary groups (*p* > 0.05). However, the FCR reached the lowest value at 0.42% Trp level. At 0.46% Trp level, CF and HSI showed the greatest values. During the feeding trial, the survival rate was above 86%, and no significant differences were observed among the treatment groups.

**Figure 1:**
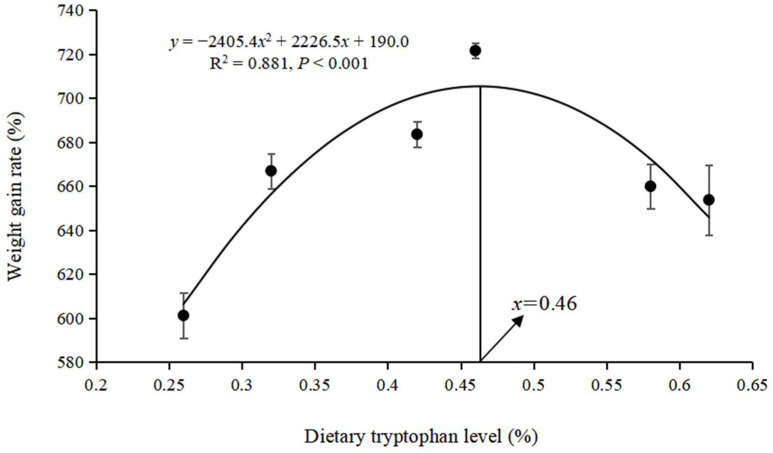
Quadratic regression curve relationship between dietary tryptophan level (%) and weight gain rate (%) of hybrid grouper.

**Figure 2:**
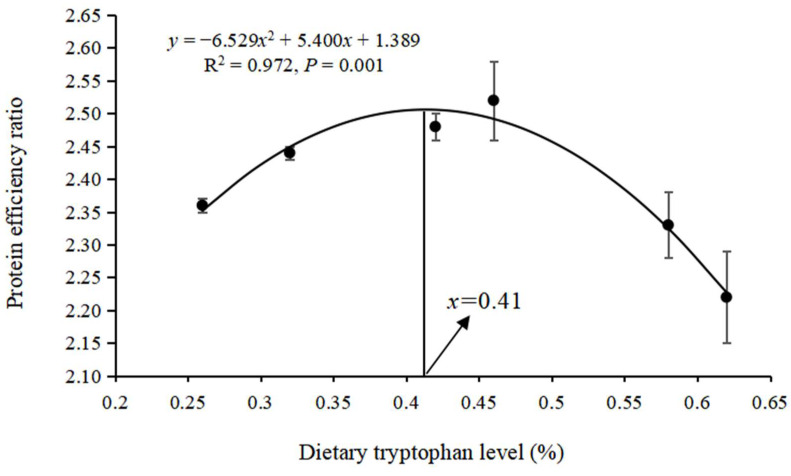
Quadratic regression curve relationship between dietary tryptophan level (%) and protein efficiency ratio of hybrid grouper.

According to Figure 1 and Figure 2, the quadratic regression curve model suggested that the optimal Trp requirements for WGR and PER in hybrid grouper were 0.46% (0.92% of dietary protein) and 0.41% (0.82% of dietary protein), respectively.

### 3.2. Proximate Composition of Fish Body

The moisture, crude protein, and crude ash contents of fish body did not differ significantly (*p* > 0.05; Table 5). The crude lipid content of fish body was considerably lower in the 0.46% Trp group compared to the 0.32% and 0.42% Trp groups (*p* < 0.05).

### 3.3. Intestinal Digestive Enzyme Activities

The intestinal amylase and lipase activities initially increased and then decreased as the dietary Trp level rose (Table 6). At a dietary Trp level of 0.46%, both intestinal trypsin and lipase activities in hybrid grouper were the highest (*p* < 0.05). Meanwhile, intestinal amylase activity was significantly higher at dietary Trp level 0.42% (*p* < 0.05).

### 3.4. Serum Biochemical Indices

Serum BUN content was significantly lower in the 0.58% Trp group compared to the other groups (*p* < 0.05; Table 7). Serum TAA content showed a trend of initially increasing and then decreasing; the highest value was observed in the 0.42% Trp group (*p* < 0.05). Serum AST activity generally increased with the increase of dietary Trp level, whereas no significant difference was observed among the 0.26%, 0.32%, 0.42%, and 0.46% Trp groups (*p* > 0.05). Serum ALT activity exhibited a trend of initially decreasing and then increasing, and the lowest value was found in the 0.46% Trp group (*p* < 0.05), while no significant difference was observed among the 0.32%, 0.42%, and 0.46% Trp groups (*p* > 0.05). Thus, the highest serum AST/ALT ratio was observed in the 0.62% Trp group, but no significant difference was found among the 0.26%, 0.32%, 0.42%, and 0.58% groups (*p* > 0.05).

### 3.5. Activities of Tryptophan Metabolism-Related Enzymes

As dietary Trp level increased, the activities of hepatic 5-HTPDC, TDO, and IDO initially enhanced and then declined; the highest values were observed at a dietary Trp level of 0.46% (*p* < 0.05; Table 8). In contrast, the hepatic TPH activity firstly decreased and subsequently increased with the increase of dietary Trp level, with the lowest value observed at dietary Trp level of 0.32% (*p* < 0.05).

### 3.6. Activities of Protein Metabolism-Related Enzymes

As dietary Trp level increased, the hepatic AST and ALT activities first depressed and then enhanced, and the lowest values were observed in the 0.42% Trp group (*p* < 0.05), whereas no significant differences were found among the 0.32%, 0.42%, and 0.46% Trp groups (*p* > 0.05; Table 9). Conversely, the hepatic GDH activity initially raised and subsequently declined with the increase of dietary Trp level; the highest value was observed in the 0.46% Trp group (*p* < 0.05). Similarly, the hepatic AMPD activity was significantly higher in the 0.46% Trp group compared to the other groups (*p* < 0.05).

### 3.7. The Relative mRNA Expression Levels of Mammalian Target of Rapamycin and Amino Acid Response Pathways in Liver

**Figure 3:**
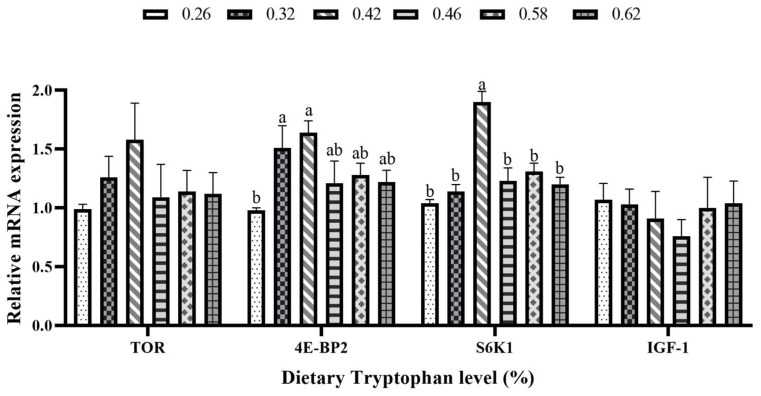
Relative mRNA expression levels of the mammalian target of rapamycin signaling pathway, including the target of rapamycin (TOR), eukaryotic initiation factor 4E-binding protein 2 (4E-BP2), S6 kinase 1 (S6K1), and insulin-like growth factor 1 (IGF-1), in the liver of juvenile hybrid grouper fed diets with various levels of tryptophan. Values are means with standard errors represented by vertical bars (*n* = 3). ^a,b^ Means with different letters were significantly different (*p* < 0.05).

The relative mRNA expression of eukaryotic initiation factor 4E-binding protein 2 (4E-BP2) in the 0.32% and 0.42% Trp groups was significantly higher than that in the control group (*p* < 0.05; Figure 3). However, the relative mRNA expression of S6 kinase 1 (S6K1) was significantly higher in the 0.42% Trp group compared to the other groups (*p* < 0.05). No significant differences in the relative mRNA expressions of target of rapamycin (TOR) and insulin-like growth factor 1 (IGF-1) were observed across the treatment groups (*p* > 0.05).

**Figure 4:**
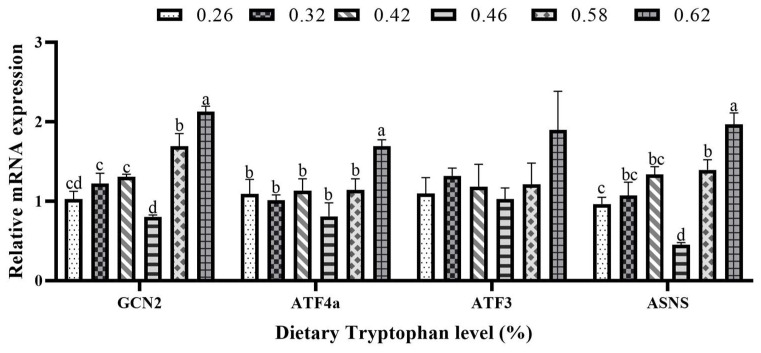
Relative mRNA expression levels of amino acid response signaling pathways, including the general control nondepressible 2 (GCN2), activating transcription factor 4a (ATF4a), activating transcription factor 3 (ATF3), and asparagine synthetase (ASNS), in the liver of juvenile hybrid grouper fed diets with various levels of tryptophan. Values are means with standard errors represented by vertical bars (*n* = 3). ^a,b,c,d^ Means with different letters were significantly different (*p* < 0.05).

The relative mRNA expression of asparagine synthetase (ASNS) was significantly lower in the 0.46% Trp group compared to the other groups (*p* < 0.05; Figure 4). Meanwhile, the relative mRNA expression level of general control nondepressible 2 (GCN2) was the lowest in the 0.46% Trp group. However, the relative mRNA expression of activating transcription factor 4a (ATF4a) was significantly higher in the 0.62% Trp group compared to the other groups (*p* < 0.05). No significant differences in the relative mRNA expression of activating transcription factor 3 (ATF3) were observed across the treatment groups (*p* > 0.05).

## 4. Discussion

A balanced amino acid profile is a crucial factor in promoting fish growth. Both deficiencies and excesses of essential amino acids can impair fish development and feed efficiency [5]. Trp is one of the essential amino acids for fish, playing a key role in their growth and development. The Trp requirement varies among different fish species, typically ranging from 0.13–0.50%. Trp deficiency in fish can lead to conditions such as scoliosis or lordosis [3,4], potentially resulting in death. However, these fish malformations can be minimized by supplementing Trp and its metabolites in diets [24]. In this study, dietary Trp supplementation significantly improved the growth performance of grouper, and those fed a diet with 0.46% Trp exhibited the highest growth performance and PER, likely due to the optimal Trp level enhancing the digestion and absorption of protein and other nutrients. According to the SOP regression model for WGR and PER, the optimal dietary lysine requirement for hybrid grouper was between 0.41% and 0.46% (0.82–0.92% of dietary protein). This requirement closely aligns with the documented Trp requirements for other species, such as Japanese flounder [7] (1.0% of dietary protein), rainbow trout [25] (0.89% of dietary protein), and African catfish (*Clarias gariepinus*) [3] (1.1% of dietary protein). It was higher than the Trp requirements for chum salmon (*Oncorhynchus keta*) [4] (0.73% of dietary protein) and red drum (*Sciaenops ocellatus*) [26] (0.80% of dietary protein). These studies indicate that variations in dietary Trp requirements are influenced by factors such as fish species, size or age, and rearing conditions.

Furthermore, body indices such as CF, VSI, and HSI are critical parameters in nutritional studies that reflect fish growth and health. In this study, dietary Trp level had no significant effect on the CF and HSI, but the VSI was increased by dietary inclusion of 0.32–0.46% Trp. The findings regarding HSI align with that reported by Kim et al. [27] in rainbow trout. However, numerous studies have shown that Trp deficiency led to an increase in HSI in fish [25,28]. The response of HSI to varying Trp levels can be difficult to interpret across different fish species, likely due to variations in dietary compositions. Overall, a hybrid grouper-fed diet with 0.46% Trp exhibited relatively increased VSI and whole-body crude protein content, suggesting that a balanced amino acid profile may enhance protein deposition in visceral organs.

In fish, the gut functions as the primary site for nutritional digestion and absorption, with digestive enzymes playing a critical role. These enzymes include trypsin, amylase, and lipase, among others. In this study, significant increases in the activities of intestinal trypsin, amylase, and lipase were observed in hybrid grouper fed diets with 0.42–0.46% Trp. These findings are consistent with studies on northern snakehead (*Channa argus*) [29], hybrid catfish (*Pelteobagrus vachelli*♀ × *Leiocassis longirostris*♂) [30], and rainbow trout [25], indicating that dietary Trp supplementation at appropriate doses enhanced the activities of digestive enzymes. Furthermore, Tang et al. [31] suggested that the observed increase in digestive enzyme activities resulting from Trp supplementation may be attributed to Trp itself and/or its metabolites. Additionally, Konturek et al. [32] found that Trp injection stimulates the secretion of cholecystokinin (CCK) in dogs. CCK is a key regulatory factor for pancreatic secretion in fish [33]. Moreover, Trp and its metabolite melatonin protect pancreatic tissue by scavenging free radicals, activating antioxidant enzymes, and regulating cytokines [34].

Protein metabolism encompasses the processes of protein synthesis and degradation within the body, involving a series of intricate biochemical reactions aimed at supplying essential amino acids and maintaining protein balance [35]. It is widely recognized that essential amino acids are crucial for protein synthesis; thus, the deficiency of any essential amino acid can hinder the protein synthesis process [36]. TOR, a crucial serine/threonine kinase, plays a central role in regulating protein synthesis. It often inhibits the rapid activation of protein synthesis induced by insulin, growth factors, and other growth-promoting agents, highlighting the significance of mTOR signaling in the activation of translational mechanisms [37]. Through phosphorylation, mTOR regulates mRNA translation via its two effectors: 4E-BP2 and S6K1 [38]. The present study showed that the relative expression levels of TOR, S6K1, and 4E-BP2 in the liver were the highest at the dietary inclusion of 0.42% Trp, although no significant difference was observed in the relative expression level of TOR among the dietary treatments. The findings indicated that suitable dietary Trp content may activate the TOR signaling pathway, thereby enhancing protein synthesis in hybrid grouper. Similarly, optimal dietary Trp levels resulted in increased expression levels of S6K1 and TOR in the livers of hybrid catfish [30]. Furthermore, adequate dietary Trp levels upregulated the relative expressions of TOR and 4E-BP1 in the muscle of rainbow trout [25]. These findings indicated that incorporating appropriate levels of Trp into the diet was essential for maintaining effective protein synthesis. Additionally, a deficiency in dietary protein or essential amino acids triggers the amino acid response (AAR) signaling pathway [39,40]. Among the regulatory enzymes that play a crucial role in the cellular response to amino acid limitation across all tissues is GCN2 [41]. Additionally, dietary amino acid deficiency may activate downstream effectors, ATF3 and ATF4. This activation will result in a decrease in protein synthesis, thereby preventing cells from initiating protein translation [42]. ASNS is a component of the GCN2 signaling pathway [43]. This study demonstrated that dietary inclusion of 0.42–0.46% Trp down-regulated the relative expressions of GCN2 and ASNS in the liver of hybrid grouper, indicating that an optimal level of Trp may inhibit the AAR signaling pathway.

Ammonia nitrogen, a by-product of protein breakdown, reflects the capacity to utilize amino acids as an energy source. A deficiency in any essential amino acid can lead to increased nitrogen production in fish [44]. Research has shown that both excess and deficiency of Trp can elevate BUN levels, potentially leading to focal tubulointerstitial injury in mice [45] and impairing protein utilization [46]. The concentration of free amino acids in blood is determined by the balance between amino acid intake from the diet and the dynamics of amino acid metabolism in the connective tissue. Free amino acids serve as the primary mode of amino acid transport between tissues, with fluctuations in their levels reflecting the organism’s protein and energy metabolism status to some extent. In this study, the lowest serum BUN level was found in hybrid grouper fed a diet with 0.58% Trp, while the highest serum TAA level was observed in fish fed a diet with 0.42% Trp, indicating a balanced amino acid profile in diets may contribute to improving protein utilization in hybrid grouper. The activities of AST and ALT in serum are commonly utilized to evaluate the liver function of fish, as these enzymes are typically released into the bloodstream during liver cell damage or injury [47]. The serum AST/ALT ratio can serve as a more reliable indicator of liver damage [48]; an obviously elevated AST/ALT ratio indicated liver injury in rats [49]. In this study, the highest serum AST/ALT ratio was found in hybrid grouper fed a diet with 0.62% Trp, suggesting that excessive dietary Trp may be detrimental to the liver health of fish.

Following the degradation of dietary proteins into amino acids, these compounds are primarily metabolized and transformed in the body through the process of deamination. Notably, transamination and deamination occur concurrently during protein metabolism. Transamination involves the transfer of an amino group from one amino acid to another compound, resulting in the synthesis of a new amino acid. In contrast, deamination involves the removal of the amino group, which is subsequently oxidized and broken down to generate energy within the body. Ultimately, this process produces urea, which is excreted from the body [50]. GDH and AMPD are essential rate-limiting enzymes in the deamination reactions of amino acids [51], while AST and ALT are the primary transaminases involved in amino acid metabolism in fish. The liver plays a critical role in protein metabolism, and the activities of deaminase and transaminases in the liver can serve as an indicator of protein metabolic status. In this study, the highest hepatic GDH and AMPD activities were found in hybrid grouper fed a diet with 0.46% Trp, while the lowest hepatic AST and ALT activities were observed in fish fed a diet with 0.42% Trp. These results suggest that dietary inclusion of 0.42% Trp may contribute to depressed amino acid transamination and thereby improve PER, whereas dietary 0.46% Trp enhanced the deamination of amino acids and thereby increased energy supply from protein. Additionally, previous research has demonstrated that excessive dietary Trp significantly elevated the hepatic ALT activity [29]; the increased hepatic AST activity was also found in hybrid grouper fed a diet with 0.62% Trp in this study, which is likely due to the transamination processes involved in the catabolism of excess amino acids [52].

Trp undergoes two primary metabolic pathways in the body: synthesis into tissue proteins and catabolism into various functional molecules. Moreover, Trp is primarily catabolized through three pathways: the kynurenine pathway, the 5-hydroxytryptamine pathway, and the pathways leading to indole derivatives [53]. Among these, the kynurenine pathway is the predominant route, accounting for approximately 95% of Trp metabolism, with the remaining Trp either decarboxylated to produce 5-HT or metabolized by microorganisms to form indole derivatives. The rate-limiting enzymes involved in the kynurenine pathway are TDO and IDO, which catalyze the oxidation of Trp to kynurenine, utilizing superoxide anion as a cofactor [54,55]. Notably, these enzymes are capable of effectively scavenging reactive oxygen species without converting them into hydrogen peroxide, demonstrating a scavenging efficiency that exceeds that of superoxide dismutase [56]. In conditions of Trp deficiency, the proteasome degrades TDO to mitigate excessive catabolism of Trp [57]. Research on the primitive vertebrate lamprey (*Petromyzontiformes*) indicates that Trp activated the IDO-kynurenine-aromatic hydrocarbon receptor pathway, thereby promoting immune tolerance [55]. In this study, dietary Trp content of 0.46% resulted in significantly elevated activities of TDO, IDO, and 5-HTPDC in the liver of hybrid grouper. These findings suggested that dietary Trp content of 0.46% not only promoted the catabolism of Trp but also enhanced the antioxidant capacity of hybrid grouper. Trp is converted to 5-hydroxytryptophan (5-HTP) by TPH1 in enterochromaffin cells of the gut or by TPH2 in neurons of the central nervous system. Subsequently, 5-HTP undergoes decarboxylation to form serotonin. An increased proportion of Trp metabolism via the 5-hydroxytryptamine pathway has been associated with conditions such as autism and irritable bowel syndrome [58]. In this study, the highest TPH activity in the liver was observed in fish fed a diet with 0.26% Trp, suggesting that dietary Trp deficiency may result in intestinal inflammation in fish.

## 5. Conclusions

According to the quadratic regression curve model, the optimal dietary Trp requirement in hybrid grouper was estimated to be 0.41–0.46% of diets (0.82–0.92% of dietary protein). This optimal level of dietary Trp not only enhanced growth performance but also improved PER by facilitating digestive enzyme activity and promoting protein synthesis.

## Figures and Tables

**Table 1:** Formulation and approximate composition (% dry matter) of the experimental diets.

	Dietary Tryptophan Level (%)					
Ingredient	0.26	0.32	0.42	0.46	0.58	0.62
Fish meal	27.00	27.00	27.00	27.00	27.00	27.00
Corn gluten meal	6.40	6.40	6.40	6.40	6.40	6.40
Brewer’s yeast meal	4.00	4.00	4.00	4.00	4.00	4.00
Wheat flour	23.38	23.38	23.38	23.38	23.38	23.38
Amino acid mixture ^1^	23.27	23.27	23.27	23.27	23.27	23.27
Guar gum	2.00	2.00	2.00	2.00	2.00	2.00
Fish oil	3.60	3.60	3.60	3.60	3.60	3.60
Soybean oil	3.70	3.70	3.70	3.70	3.70	3.70
Soybean phospholipid	2.00	2.00	2.00	2.00	2.00	2.00
Ca(H_2_PO_4_)_2_	1.50	1.50	1.50	1.50	1.50	1.50
Choline chloride	0.50	0.50	0.50	0.50	0.50	0.50
Vitamin C	0.02	0.02	0.02	0.02	0.02	0.02
Ethoxyquin	0.03	0.03	0.03	0.03	0.03	0.03
Y_2_O_3_	0.05	0.05	0.05	0.05	0.05	0.05
Coated L-tryptophan (90.3%) ^2^	0.00	0.11	0.22	0.33	0.44	0.55
Coated L-alanine (90.3%) ^2^	0.55	0.44	0.33	0.22	0.11	0.00
Vitamin premix ^3^	1.00	1.00	1.00	1.00	1.00	1.00
Mineral premix ^3^	1.00	1.00	1.00	1.00	1.00	1.00
Proximate composition						
Dry matter (% DM)	89.86	88.76	88.29	88.98	88.65	90.65
Crude protein (% DM)	49.98	49.13	49.85	49.38	49.53	50.38
Crude lipid (% DM)	12.04	12.62	12.39	12.00	12.31	12.34
Crude ash (% DM)	8.02	8.09	8.08	8.04	7.93	8.11

^1^ Crystalline amino acids mixture (% of diet) included: methionine, 0.30; lysine, 1.96; threonine, 0.70; arginine, 3.38; isoleucine, 0.48; leucine, 0.69; valine, 0.51; histidine, 0.08; phenylalanine, 0.29; cystine, 0.12; glycine, 3.37; serine, 0.59; alanine, 1.63; proline, 2.27; aspartic acid, 2.52; glutamic acid, 1.89; tyrosine, 0.17. ^2^ Supplied by Shanxi Baichuan Kangze Biotechnology Co., Ltd., Xianyang, China, Purity 99.9%. ^3^ Supplied by Qingdao Master Bio-Tech Co., Ltd., Qingdao, China. Vitamin premix (g/kg of mixture) included 1.55 g retinyl acetate (2,800,000 IU/g), 0.03 g cholecalciferol, 40 g DL-α-tocopheryl acetate, 8 g menadione, 6 g thiamine hydrochloride, 9 g riboflavin, 7 g pyridoxine hydrochloride, 0.05 g vitamin B_12_, 50 g ascorbic acid, 30 g calcium D-pantothenate, 45 g niacin, 2.5 g folic acid, 0.1 g D-biotin, and 100 g inositol; mineral premix (g/kg of mixture) included 186 g FeSO_4_⋅7H_2_O, 53 g ZnSO_4_⋅7H_2_O, 25 g MnSO_4_⋅H_2_O, 2.7 g CuSO_4_⋅5H_2_O, 0.4 g CoCl_2_⋅6H_2_O, 0.1 g Na_2_SeO_3_, and 0.13 g KI.

**Table 2:** Amino acid profile of the experimental diets (% dry matter).

	Dietary Tryptophan Level (%)					
	0.26	0.32	0.42	0.46	0.58	0.62
Essential amino acids (EAA)						
Tryptophan	0.26	0.32	0.42	0.46	0.58	0.62
Methionine	1.11	1.08	1.07	1.05	1.07	1.16
Lysine	3.01	2.92	2.94	2.99	2.94	2.99
Threonine	1.90	1.87	1.70	1.73	1.87	1.94
Isoleucine	1.67	1.60	1.64	1.66	1.64	1.68
Leucine	3.14	3.10	3.05	3.10	3.16	3.22
Phenylalanine	1.64	1.60	1.62	1.65	1.66	1.68
Arginine	2.60	2.51	2.51	2.59	2.52	2.60
Histidine	0.85	0.77	0.77	0.81	0.80	0.80
Valine	1.74	1.69	1.77	1.76	1.73	1.72
∑EAA	17.92	17.46	17.49	17.80	17.97	18.41
Non-essential amino acids (NEAA)						
Aspartate	5.47	5.33	5.28	5.36	5.44	5.62
Glutamate	7.31	7.20	7.01	7.13	7.22	7.44
Glycine	3.85	3.76	3.71	3.96	3.83	3.88
Alanine	3.49	3.34	3.25	3.21	3.13	3.09
Cystine	0.52	0.52	0.54	0.57	0.50	0.55
Serine	1.62	1.60	1.33	1.31	1.64	1.70
Proline	4.11	4.05	4.06	3.98	5.04	4.39
Tyrosine	1.26	1.21	1.18	1.22	1.14	1.19
∑NEAA	27.63	27.01	26.36	26.74	27.94	27.86

**Table 3:** Primers used for quantitative RT-PCR (qPCR).

Genes	Forward and Reverse Primer Sequences (5’-3’)	Efficiency of Primers (%)	Genbank Accession No.
TOR	F: CAAGGTTTCTTCCGCTCCATCTCC R: CTCCACCAGGGCTTCATTCACTTC	93.80	JN850959.1
S6K1	F: TCACCTCCCGCACTCCTAAAGAC R: GCTTGACCTTCTCCACTGAACCTTC	103.54	XM_033643204.1
4E-BP2	F: TAACTCTCCCATTGCCCAGACTCC R: GTGGTTGTTGGCTTCGTTCTTCTTG	109.18	XM_033613907.1
GCN2	F: AGGAGGACTGTCTCGTGGTGAAC R: GAGTGTGGTTGGTGAGGCTTTGG	100.71	XM_042500953.1
ATF3	F: CCAAACACCCGAGGATGAGAGAAAC R: AGGAGGAGGCGGAGGAGGAG	107.71	XM_042503803.1
ATF4a	F: TGGAGCAGACGATGGCAAAGATG R: CGGATGAGCAGGAACCAATGAGG	104.66	XM_042485343.1
ASNS	F: GCTCCATCTGTATGACCGCTTTG R: GCAAGGAATCCATCGTCTGTGAG	98.03	XM_033636649.1
IGF-1	F: TGCGGCGTCTGGAGATGTAC R: AGGTAAAGGTCTCTTGGGTGCTC	94.92	XM_033614181.1
β-actin	F: TTCACCACCACAGCCGAGAGG R: GAGGAGGAGGAGGCAGCAGTG	90.94	AY510710.2

TOR = target of rapamycin; S6K1 = S6 kinase 1; 4E-BP2 = eukaryotic initiation factor 4E-binding protein 2; GCN2 = general control nondepressible 2; ATF3 = activating transcription factor 3; ATF4a = activating transcription factor 4a; ASNS = asparagine synthetase; IGF-1 = insulin-like growth factor 1.

**Table 4:** Growth performance and body indexes of juvenile hybrid grouper fed diets with various levels of tryptophan.

Dietary Tryptophan Level (%)	FBW (g)	WGR (%)	SGR (%/d)	FIR (%)	FCR	PER	SR (%)	CF (g/cm^3^)	VSI (%)	HSI (%)
0.26	73.59 ± 0.62 ^d^	601.21 ± 5.89 ^d^	2.87 ± 0.01 ^d^	1.89 ± 0.00	0.86 ± 0.00	2.36 ± 0.01 ^bc^	86.66 ± 3.84	2.89 ± 0.02	10.51 ± 0.65 ^b^	3.94 ± 0.08
0.32	80.49 ± 0.83 ^bc^	666.91 ± 7.91 ^bc^	2.98 ± 0.01 ^bc^	1.87 ± 0.00	0.83 ± 0.00	2.44 ± 0.01 ^ab^	87.77 ± 2.22	2.92 ± 0.04	11.97 ± 0.19 ^a^	3.97 ± 0.27
0.42	82.24 ± 0.61 ^b^	683.53 ± 5.84 ^b^	3.01 ± 0.00 ^b^	1.84 ± 0.00	0.80 ± 0.00	2.48 ± 0.02 ^ab^	85.55 ± 2.22	2.98 ± 0.04	12.38 ± 0.49 ^a^	4.17 ± 0.31
0.46	86.23 ± 0.37 ^a^	721.53 ± 3.54 ^a^	3.10 ± 0.00 ^a^	1.87 ± 0.06	0.85 ± 0.03	2.52 ± 0.06 ^a^	86.66 ± 5.08	3.01 ± 0.03	12.90 ± 0.28 ^a^	4.62 ± 0.30
0.58	79.75 ± 0.60 ^c^	659.88 ± 5.73 ^c^	2.98 ± 0.01 ^bc^	1.95 ± 0.04	0.87 ± 0.01	2.33 ± 0.05 ^bc^	86.66 ± 1.92	2.93 ± 0.04	11.69 ± 0.34 ^ab^	4.01 ± 0.14
0.62	79.11 ± 0.97 ^c^	653.71 ± 9.21 ^c^	2.97 ± 0.01 ^c^	1.87 ± 0.01	0.89 ± 0.03	2.22 ± 0.07 ^c^	89.99 ± 3.33	2.86 ± 0.02	11.64 ± 0.17 ^ab^	4.01 ± 0.13
ANOVA										
*p*-value	<0.001	<0.001	<0.001	0.334	0.168	0.010	0.949	0.151	0.020	0.341
Regression										
Model	SOP	SOP	SOP	/	/	SOP	/	/	SOP	/
*p*-value	<0.001	<0.001	<0.001	/	/	<0.001	/	/	0.002	/
Adj. R^2^	0.802	0.802	0.769	/	/	0.613	/	/	0.513	/
OPTI INCL	0.463	0.463	0.465	/	/	0.414	/	/	0.461	/

Values are means ± standard error (SE) of three replications. Means with different superscript letter(s) in the same column are significantly different (*p* < 0.05). SOP = second-order polynomial trend; Adj. R^2^ = adjusted R square; OPTI INCL = optimal inclusion level of tryptophan; FBW = final body weight; WGR = weight gain rate; SGR = specific growth rate; FIR = feed intake ratio; FCR = feed conversion ratio; PER = protein efficiency ratio; SR = survival rate; CF = condition factor; VSI = viscerosomatic index; HSI = hepatosomatic index.

**Table 5:** Whole-body composition of juvenile hybrid grouper fed diets with various levels of tryptophan.

Dietary Tryptophan Level (%)	Moisture (%)	Crude Protein (%)	Crude Lipid (%)	Crude Ash (%)
0.26	72.68 ± 0.42	59.53 ± 0.58	25.21 ± 0.21 ^ab^	14.53 ± 0.06
0.32	72.65 ± 0.31	60.16 ± 0.67	25.51 ± 0.14 ^a^	13.91 ± 0.39
0.42	72.32 ± 0.17	61.80 ± 1.76	25.48 ± 0.11 ^a^	14.30 ± 0.16
0.46	72.37 ± 0.33	61.73 ± 2.71	24.66 ± 0.18 ^b^	14.33 ± 0.16
0.58	72.93 ± 0.22	59.99 ± 1.59	25.18 ± 0.18 ^ab^	14.15 ± 0.19
0.62	72.66 ± 0.33	56.56 ± 1.08	25.22 ± 0.16 ^ab^	14.09 ± 0.26
ANOVA				
*p*-value	0.760	0.272	0.049	0.544
Regression				
Model	/	/	NR	/
*p*-value	/	/	0.637	/
Adj. R^2^	/	/	/	/
OPTI INCL	/	/	/	/

Values are means ± standard error (SE) of three replications. Means with different superscript letter(s) in the same column are significantly different (*p* < 0.05). NR = no relationship; Adj. R^2^ = adjusted R square; OPTI INCL = optimal inclusion level of tryptophan.

**Table 6:** Intestinal digestive enzyme activities of juvenile hybrid grouper fed diets with various levels of tryptophan.

Dietary Tryptophan Level (%)	Trypsin (U/mg Protein)	Lipase (U/g Protein)	Amylase (U/mg Protein)
0.26	277.22 ± 28.14 ^b^	2.00 ± 0.14 ^b^	0.51 ± 0.00 ^d^
0.32	237.24 ± 32.52 ^b^	2.46 ± 0.12 ^b^	0.55 ± 0.00 ^c^
0.42	213.78 ± 3.30 ^b^	2.94 ± 0.21 ^ab^	0.83 ± 0.01 ^a^
0.46	445.40 ± 3.95 ^a^	3.44 ± 0.35 ^a^	0.67 ± 0.01 ^b^
0.58	272.04 ± 14.13 ^b^	2.71 ± 0.46 ^ab^	0.42 ± 0.00 ^e^
0.62	286.19 ± 39.29 ^b^	2.22 ± 0.25 ^b^	0.36 ± 0.00 ^f^
ANOVA			
*p*-value	<0.001	0.043	<0.001
Regression			
Model	NR	SOP	SOP
*p*-value	0.523	0.004	<0.001
Adj. R^2^	/	0.457	0.798
OPTI INCL	/	0.456	0.418

Values are means ± standard error (SE) of three replications. Means with different superscript letter(s) in the same column are significantly different (*p* < 0.05). NR = no relationship; SOP = second-order polynomial trend; Adj. R^2^ = adjusted R square; OPTI INCL = optimal inclusion level of tryptophan.

**Table 7:** Biochemical parameters in serum of hybrid grouper fed diets with different levels of tryptophan.

Dietary Tryptophan Level (%)	BUN (nmol/L)	TAA (μmol/mL)	AST (U/L)	ALT (U/L)	AST/ALT
0.26	61.47 ± 4.82 ^a^	26.76 ± 1.01 ^cd^	359.14 ± 21.06 ^c^	754.59 ± 25.71 ^a^	0.47 ± 0.03 ^c^
0.32	61.47 ± 4.32 ^a^	29.05 ± 0.93 ^bc^	402.39 ± 44.09 ^bc^	507.33 ± 33.87 ^d^	0.81 ± 0.13 ^bc^
0.42	68.39 ± 0.86 ^a^	41.02 ± 0.64 ^a^	412.71 ± 40.91 ^bc^	552.72 ± 58.35 ^cd^	0.76 ± 0.12 ^bc^
0.46	65.80 ± 1.73 ^a^	32.05 ± 2.06 ^b^	430.01 ± 55.36 ^bc^	480.99 ± 39.84 ^d^	0.91 ± 0.18 ^b^
0.58	42.42 ± 2.29 ^b^	23.93 ± 2.03 ^d^	507.06 ± 30.28 ^b^	665.48 ± 4.04 ^ab^	0.76 ± 0.04 ^bc^
0.62	59.50 ± 3.97 ^a^	23.71 ± 1.28 ^d^	1002.93 ± 41.21 ^a^	640.51 ± 16.46 ^bc^	1.57 ± 0.10 ^a^
ANOVA					
*p*-value	0.002	<0.001	<0.001	0.001	0.001
Regression					
Model	NR	SOP	L	SOP	L
*p*-value	0.087	<0.001	0.001	0.003	0.003
Adj. R^2^	/	0.599	0.503	0.476	0.407
OPTI INCL	/	0.423	/	0.445	/

Values are means ± standard error (SE) of three replications. Means with different superscript letter(s) in the same column are significantly different (*p* < 0.05). NR = no relationship; L = linear trend; SOP = second-order polynomial trend; Adj. R^2^ = adjusted R square; OPTI INCL = optimal inclusion level of tryptophan. BUN = blood urea nitrogen; TAA = total amino acids; AST = aspartate aminotransferase; ALT = alanine aminotransferase.

**Table 8:** Tryptophan metabolism-related parameters in the liver of hybrid grouper fed diets with different levels of tryptophan.

Dietary Tryptophan Level (%)	5-HTPDC (U/mg Protein)	TPH (U/mg Protein)	TDO (U/mg Protein)	IDO (IU/mg Protein)
0.26	152.73 ± 3.10 ^b^	77.68 ± 1.66 ^a^	25.98 ± 0.15 ^d^	17.41 ± 0.06 ^b^
0.32	155.79 ± 5.98 ^b^	33.08 ± 1.32 ^e^	29.33 ± 1.48 ^c^	17.06 ± 0.22 ^b^
0.42	156.82 ± 5.62 ^b^	40.03 ± 0.57 ^d^	37.69 ± 0.88 ^b^	16.73 ± 0.29 ^b^
0.46	284.66 ± 7.70 ^a^	43.12 ± 1.86 ^d^	41.30 ± 0.66 ^a^	25.85 ± 0.38 ^a^
0.58	119.22 ± 5.69 ^c^	52.35 ± 2.05 ^c^	39.90 ± 0.78 ^ab^	11.84 ± 0.21 ^c^
0.62	128.63 ± 3.93 ^c^	57.24 ± 0.97 ^b^	31.76 ± 1.44 ^c^	8.25 ± 0.27 ^d^
ANOVA				
*p*-value	<0.001	<0.001	<0.001	<0.001
Regression				
Model	SOP	SOP	SOP	SOP
*p*-value	0.023	0.001	<0.001	<0.001
Adj. R^2^	0.314	0.540	0.794	0.622
OPTI INCL	0.429	0.451	0.483	0.402

Values are means ± standard error (SE) of three replications. Means with different superscript letter(s) in the same column are significantly different (*p* < 0.05). SOP = second-order polynomial trend; Adj. R^2^ = adjusted R square; OPTI INCL = optimal inclusion level of tryptophan. 5-HTPDC = 5-hydroxytryptophan decarboxylase; IDO = indoleamine 2,3-dioxygenase; TDO = tryptophan-2,3-dioxygenase; TPH = tryptophan hydroxylase.

**Table 9:** Protein metabolism-related parameters in the liver of hybrid grouper fed diets with different levels of tryptophan.

Dietary Tryptophan Level (%)	AST (U/g Protein)	ALT (U/g Protein)	AMPD (U/mg Protein)	GDH (U/g Protein)
0.26	15.54 ± 1.25 ^b^	16.80 ± 0.71 ^a^	43.22 ± 0.94 ^b^	19.12 ± 1.09 ^c^
0.32	9.22 ± 0.60 ^c^	11.79 ± 0.99 ^bc^	41.81 ± 2.73 ^b^	21.52 ± 0.88 ^c^
0.42	8.90 ± 0.23 ^c^	9.59 ± 0.50 ^c^	38.12 ± 0.96 ^b^	27.19 ± 0.85 ^b^
0.46	12.85 ± 1.00 ^bc^	11.72 ± 1.07 ^bc^	98.20 ± 2.51 ^a^	30.52 ± 1.31 ^a^
0.58	16.66 ± 0.72 ^b^	13.59 ± 0.58 ^b^	43.60 ± 1.53 ^b^	19.80 ± 0.52 ^c^
0.62	21.83 ± 2.83 ^a^	12.81 ± 0.87 ^b^	39.10 ± 1.87 ^b^	11.83 ± 0.55 ^d^
ANOVA				
*p*-value	<0.001	0.001	<0.001	<0.001
Regression				
Model	SOP	SOP	NR	SOP
*p*-value	<0.001	0.002	0.098	<0.001
Adj. R^2^	0.722	0.510	/	0.851
OPTI INCL	0.399	0.460	/	0.425

Values are means ± standard error (SE) of three replications. Means with different superscript letter(s) in the same column are significantly different (*p* < 0.05). NR = no relationship; SOP = second-order polynomial trend; Adj. R^2^ = adjusted R square; OPTI INCL = optimal inclusion level of tryptophan. AST = aspartate aminotransferase; ALT = alanine aminotransferase; AMPD = adenosine monophosphate deaminase; GDH = glutamate dehydrogenase.

## Data Availability

The data that support the findings of this study are available on request from the corresponding author.
